# A Frobenius Norm Regularization Method for Convolutional Kernel Tensors in Neural Networks

**DOI:** 10.1155/2022/3277730

**Published:** 2022-08-25

**Authors:** Pei-Chang Guo

**Affiliations:** School of Science, China University of Geosciences, Beijing 100083, China

## Abstract

The convolutional neural network is a very important model of deep learning. It can help avoid the exploding/vanishing gradient problem and improve the generalizability of a neural network if the singular values of the Jacobian of a layer are bounded around 1 in the training process. We propose a new Frobenius norm penalty function for a convolutional kernel tensor to let the singular values of the corresponding transformation matrix be bounded around 1. We show how to carry out the gradient-type methods. This provides a potentially useful regularization method for the weights of convolutional layers.

## 1. Introduction

In recent years, deep convolutional neural networks have been applied successfully in many fields, such as face recognition, self-driving cars, natural language understanding, and speech recognition [1]. As we know, in the field of deep learning, convolution without the flip is very important arithmetic [2]. From the viewpoint of linear algebra, each convolutional kernel corresponds to a linear transformation matrix. Given the input *X* and a kernel *K*, the output of convolution *Y*=*K∗X* can be reshaped from the matrix-vector multiplication of a corresponding transformation matrix *M* with the reshaped *X*. We use vec(*X*) to denote the vectorization of *X*. If *X* is a matrix, vec(*X*) is the column vector got by stacking the columns of *X* on top of one another. If *X* is a tensor, vec(*X*) is the column vector got by stacking the columns of the flattening of *X* along the first index (see [3] for more on flattening of a tensor). Thus, given a kernel *K*, assume *M* is the linear transformation matrix corresponding to the kernel *K*, we have

(1) $$\begin{array}{c} \text{vec}\left(Y\right)=M\text{vec}\left(X\right). \end{array}$$

Training a neural network can be seen as an optimization problem, which is seeking the optimal weights (parameters) by reaching the minimum of loss function on the training data. Exploding and vanishing gradients are fundamental obstacles to effectively training a deep neural network [4]. The singular values of the Jacobian of a layer bound the factor by which it changes the norm of the backpropagated signal. On the other hand, the generalizability of a model can be improved by reducing the sensitivity of a loss function against the input data perturbation [5–7]. Therefore, when training deep convolutional neural networks, if the singular values of the Jacobian of each layer are close to 1, it can help to avoid the exploding/vanishing gradient problem [4, 8–10] and improve the generalizability of a neural network [7, 11, 12]. This is to let the singular values of each linear transformation matrix *M* be bounded around 1. Our contribution in this paper is to give a Frobenius norm penalty function, to penalize the kernel *K* such that the singular values of the corresponding transformation matrix *M* be bounded around 1, thus ‖vec(*Y*)‖ ≈ ‖vec(*X*)‖, where ‖·‖ denotes a certain vector norm.

First, we briefly introduce the convolution arithmetic in deep learning; please see reference [2] for details. Depending on different strides and padding patterns, there are many different forms of convolution arithmetic [2]. Without losing generality, in this paper we will consider the same convolution with unit strides. The notation *∗* is to denote the convolution arithmetic in deep learning and · is to round a number to the nearest integer greater than or equal to that number. If a convolutional kernel is a matrix *K* ∈ *ℝ*^*k*×*k*^ and the input is a matrix *X* ∈ *ℝ*^*N*×*N*^, each entry of the output *Y* ∈ *ℝ*^*N*×*N*^ is produced by

(2) $$\begin{array}{c} Y_{r,s}={\left(K*X\right)}_{r,s}=\sum_{p\in \left\{1,\ldots ,k\right\}}\sum_{q\in \left\{1,\ldots ,k\right\}}X_{r-m+p,s-m+q}K_{p,q}, \end{array}$$

where m=k/2 and *X*_*i*,*j*_=0 if *i* ≤ 0 or *i* > *N*, or *j* ≤ 0 or *j* > *N*.

In convolutional neural networks, usually there are multichannels and a convolutional kernel is represented by a 4-dimensional tensor. If a convolutional kernel is a 4-dimensional tensor *K* ∈ *ℝ*^*k*×*k*×*g*×*h*^ and the input is 3-dimensional tensor *X* ∈ *ℝ*^*N*×*N*×*g*^, each entry of the output *Y* ∈ *ℝ*^*N*×*N*×*h*^ is produced by

(3) $$\begin{array}{c} Y_{r,s,c}={\left(K*X\right)}_{r,s,c}=\sum_{d\in \left\{1,\ldots ,g\right\}}\sum_{p\in \left\{1,\ldots ,k\right\}}\sum_{q\in \left\{1,\ldots ,k\right\}}X_{r-m+p,s-m+q,d}K_{p,q,d,c}, \end{array}$$

where m=k/2 and *X*_*i*,*j*,*d*_=0 if *i* ≤ 0 or *i* > *N*, or *j* ≤ 0 or *j* > *N*. When we refer to the convolution arithmetic in deep learning, only element-wise multiplication and addition are performed and there is no reverse for the convolutional kernel in deep learning.

Given a general kernel *K* whose size is *k* × *k* × *g* × *h* and the input data matrix size *N* × *N* × *g*, and assume *M* is the linear transformation matrix corresponding to the kernel *K*, we will give a regularization term to let the following term toward a smaller value.

(4) $$\begin{array}{c} \max\left\{\left|\sigma_{\max}\left(M\right)-1\right|,\left|\sigma_{\min}\left(M\right)-1\right|\right\}. \end{array}$$

But the above term (4) is hard to minimize directly. In this paper, we will use ‖*M*^*T*^*M* − *I*‖_*F*_^2^ as the penalty function, where ‖·‖_*F*_ denotes the Frobenius norm of a matrix and *I* is the identity matrix, to let the singular values of *M* be bounded around 1.

In this paper, we will show how to modify the entries of the kernel *K* to minimize ‖*M*^*T*^*M* − *I*‖_*F*_^2^ from the viewpoint of linear algebra. The knowledge in the field of matrix analysis plays a key role in this paper. As we know, given a matrix, the singular values/eigenvalues are continuous functions depending on the entries of the matrix [13]. We can calculate the partial derivatives of a singular value with respect to the entries, and let the singular values of a matrix be changed by modifying the entries. Here the transformation matrix *M* corresponding to a convolutional kernel is structured, i.e., *M* has a special matrix structure. Our goal is to regularize the singular values of *M* through modifying entries of the kernel *K*. The modification of *K* is actually to carry out a modification of *M* on a special matrix manifold. The contribution is that we derive a mathematical formula for the gradient of ‖*M*^*T*^*M* − *I*‖_*F*_^2^ with respect to the kernel *K*, i.e., *∂*‖*M*^*T*^*M* − *I*‖_*F*_^2^/*∂k*_*p*,*q*,*z*,*y*_. Then gradient-based algorithms can be applied to effectively let the singular values of convolutional layers be bounded. Compared with the 2 norm, the Frobenius norm of a matrix is less sensitive to the perturbations of matrix entries. This Frobenius norm-based formula will be numerically stable.

There are many techniques from different perspectives to improve the performance of a neural network model. In [14], a semisupervised deep model, which is robust over imbalanced and small training data sets, is proposed for human activity recognition from multimodal wearable sensory data. A semisupervised feature selection method, which shows superiority in video semantic recognition-related tasks, is proposed from the perspective that the instances with similar labels should have a larger probability of being neighbors [15]. Two deep learning-based frameworks are proposed, which make sense of spatio-temporal preserving representations for electroencephalography-based human intension recognition [16]. A modeling method based on neural networks describes the hysteresis nonlinearity better by adding a nonlinear function in the input layer [17].

As we know, batch normalization and dropout are two popular regularization methods [18–21]. Recently, for the weights of a neural network, there have been many papers devoted to enforcing the orthogonality or spectral norm regularization [8, 9, 12, 22]. The difference between our paper and papers including [8, 9, 12, 22] and the references therein is about how to handle convolutions. They enforce the constraint directly on the *h* × (*gkk*) matrix reshaped from the kernel *K* ∈ *ℝ*^*k*×*k*×*g*×*h*^, while we enforce the constraint on the transformation matrix *M* corresponding to the convolution kernel *K*. In [10], a convolutional layer is projected onto the set of layers obeying a bound on the operator norm of the layer and this is shown to be an effective regularizer. A drawback of the method in [10] is that projection can prevent the singular values of the transformation matrix from being large but cannot avoid the singular values to be too small. In [23], a 2-norm regularization method is proposed for convolutional kernels, but it is not a stable algorithm because the largest singular value may be overtaken by the second or the third largest singular value after one updating. In this paper, we propose a Frobenius norm regularization method for convolutional kernels.

As we have mentioned, the input channels and the output channels may be more than one, so the kernel is usually represented by a tensor *K* ∈ *ℝ*^*k*×*k*×*g*×*h*^. The rest of the paper is organized as follows: In Section 2, we first consider the case that the numbers of input channels and the output channels are both 1. We propose the penalty function, calculate the partial derivatives, and propose the gradient descent algorithm for this case. In Section 3, we propose the penalty function and calculate the partial derivatives for the case of multichannel convolution. In Section 4, we present numerical results to show the method is feasible and effective. In Section 5, we will give some conclusions and point out some interesting work that could be done in the future.

## 2. Penalty Function for One-Channel Convolution

As a warm-up, we first focus on the case that the numbers of input channels and the output channels are both 1. In this case, the weights of the kernel are a *k* × *k* matrix. Without loss of generality, assuming the data matrix is *N* × *N*, we use a 3 × 3 matrix as a convolution kernel to show the associated transformation matrix. Let *K* be the convolution kernel:

(5) $$\begin{array}{c} K=\left(\begin{array}{ccc} k_{11} & k_{12} & k_{13}\\ k_{21} & k_{22} & k_{23}\\ k_{31} & k_{32} & k_{33} \end{array}\right). \end{array}$$

Then the transformation matrix corresponding with the convolution arithmetic is

(6) $$\begin{array}{c} M=\left(\begin{array}{cccccc} A_{0} & A_{-1} & 0 & 0 & \cdots & 0\\ A_{1} & A_{0} & A_{-1} & \ddots & \ddots & \vdots \\ 0 & A_{1} & A_{0} & \ddots & \ddots & 0\\ 0 & \ddots & \ddots & \ddots & A_{-1} & 0\\ \vdots & \ddots & \ddots & A_{1} & A_{0} & A_{-1}\\ 0 & \cdots & 0 & 0 & A_{1} & A_{0} \end{array}\right), \end{array}$$

where

(7) $$\begin{array}{c} A_{0}=\left(\begin{array}{cccccc} k_{22} & k_{32} & 0 & 0 & \cdots & 0\\ k_{12} & k_{22} & k_{32} & \ddots & \ddots & \vdots \\ 0 & k_{12} & k_{22} & \ddots & \ddots & 0\\ 0 & \ddots & \ddots & \ddots & k_{32} & 0\\ \vdots & \ddots & \ddots & k_{12} & k_{22} & k_{32}\\ 0 & \cdots & 0 & 0 & k_{12} & k_{22} \end{array}\right),\\ A_{-1}=\left(\begin{array}{cccccc} k_{23} & k_{33} & 0 & 0 & \cdots & 0\\ k_{13} & k_{23} & k_{33} & \ddots & \ddots & \vdots \\ 0 & k_{13} & k_{23} & \ddots & \ddots & 0\\ 0 & \ddots & \ddots & \ddots & k_{33} & 0\\ \vdots & \ddots & \ddots & k_{13} & k_{23} & k_{33}\\ 0 & \cdots & 0 & 0 & k_{13} & k_{23} \end{array}\right),\\ A_{1}=\left(\begin{array}{cccccc} k_{21} & k_{31} & 0 & 0 & \cdots & 0\\ k_{11} & k_{21} & k_{31} & \ddots & \ddots & \vdots \\ 0 & k_{11} & k_{21} & \ddots & \ddots & 0\\ 0 & \ddots & \ddots & \ddots & k_{31} & 0\\ \vdots & \ddots & \ddots & k_{11} & k_{21} & k_{31}\\ 0 & \cdots & 0 & 0 & k_{11} & k_{21} \end{array}\right). \end{array}$$

In this case, the transformation matrix *M* corresponding to the convolutional kernel *K* is a *N*^2^ × *N*^2^ doubly block banded Toeplitz matrix, i.e., a block banded Toeplitz matrix with its blocks are banded Toeplitz matrices. For the details about Toeplitz matrices, please see references [24, 25]. We will let *n*=*N*^2^ and use *𝒯* to denote the set of all matrices like *M* in (6), i.e., doubly block banded Toeplitz matrices with the fixed band.

We will use ‖*M*^*T*^*M* − *I*‖_*F*_^2^ as the penalty function to regularize the convolutional kernel *K* and calculate *∂*‖*M*^*T*^*M* − *I*‖_*F*_^2^/*∂K*_*p*,*q*_, i.e., the partial derivative of Frobenius norm of *M*^*T*^*M* − *I* versus each entry *K*_*p*,*q*_ of the convolution kernel. Our method provides a new method to calculate the gradient of the penalty function of the transformation matrix versus the convolution kernel. People can construct other penalty functions of *M* and get the gradient descent method when training their convolutional networks. The following lemma is easy but useful in the following derivation.

**Lemma 1 .**

The partial derivative of square of Frobenius norm of *A* ∈ *ℝ*^*n*×*n*^ with respect to each entry *a*_*ij*_ is *∂*‖*A*‖_*F*_^2^/*∂a*_*ij*_=2*a*_*ij*_.

If an entry *a*_*ij*_ of the matrix *A* ∈ *ℝ*^*n*×*n*^ changes, only the entries belonging to *j*-th row or *j*-th volume of the matrix *A*^*T*^*A* are affected. Actually, we have the following lemma.

**Lemma 2 .**

If we use (*A*^*T*^*A*)_*s*,*t*_ to denote the (*s*, *t*) entry of the matrix *A*^*T*^*A*, then *∂*(*A*^*T*^*A*)_*s*,*t*_/*∂a*_*ij*_ is the (*s*, *t*) entry of the matrix *D*=*A*^*T*^(*e*_*i*_*e*_*j*_^*T*^)+(*e*_*j*_*e*_*i*_^*T*^)*A*, where

(8) $$\begin{array}{c} D=\left(\begin{array}{cccccccc} 0 & \cdots & \cdots & 0 & a_{i1} & 0 & \cdots & 0\\ \vdots & \vdots & \vdots & \vdots & a_{i2} & \vdots & \vdots & \vdots \\ \vdots & \vdots & \vdots & \vdots & \vdots & \vdots & \vdots & \vdots \\ 0 & \cdots & \cdots & 0 & a_{i,j-1} & 0 & \cdots & 0\\ a_{i1} & a_{i2} & \cdots & a_{i,j-1} & 2a_{ij} & a_{i,j+1} & \cdots & a_{in}\\ 0 & \cdots & \cdots & 0 & a_{i,j+1} & 0 & \cdots & 0\\ \vdots & \vdots & \vdots & \vdots & \vdots & \vdots & \vdots & \vdots \\ 0 & \cdots & \cdots & 0 & a_{in} & 0 & \cdots & 0 \end{array}\right). \end{array}$$

We have the following formula from Lemma 1 and Lemma 2

(9) $$\begin{array}{c} \frac{1}{2}\frac{\partial {\left\|M^{T}M-I\right\|}_{F}^{2}}{\partial m_{ij}}=\frac{1}{2}\sum_{s,t=1,\ldots ,n}\frac{\partial {\left\|M^{T}M-I\right\|}_{F}^{2}}{\partial {\left(M^{T}M-I\right)}_{s,t}}\frac{\partial {\left(M^{T}M-I\right)}_{s,t}}{\partial m_{ij}}\\ =\sum_{t=1,\ldots ,n}{\left(M^{T}M-I\right)}_{j,t}m_{it}+\sum_{s=1,\ldots ,n}{\left(M^{T}M-I\right)}_{s,j}m_{is}. \end{array}$$

For a matrix *M* ∈ *𝒯*, the value of *K*_*p*,*q*_ will appear in different (*i*, *j*) indexes. We use Ω to denote this index set, i.e., for each (*i*, *j*) ∈ Ω , we have *m*_*ij*_=*K*_*p*,*q*_. The chain rule formula about the derivative tells us that, if we want to calculate *∂*‖*M*^*T*^*M* − *I*‖_*F*_^2^/*∂K*_*p*,*q*_, we should calculate *∂*‖*M*^*T*^*M* − *I*‖_*F*_^2^/*∂m*_*ij*_ for all (*i*, *j*) ∈ Ω and take the sum, i.e.,

(10) $$\begin{array}{c} \frac{1}{2}\frac{\partial {\left\|M^{T}M-I\right\|}_{F}^{2}}{\partial K_{p,q}}=\frac{1}{2}\sum_{\left(i,j\right)\in \Omega }\frac{\partial {\left\|M^{T}M-I\right\|}_{F}^{2}}{\partial m_{ij}}\\ =\sum_{\left(i,j\right)\in \Omega }\left(\sum_{t=1,\ldots ,n}{\left(M^{T}M-I\right)}_{j,t}m_{it}+\sum_{s=1,\ldots ,n}{\left(M^{T}M-I\right)}_{s,j}m_{is}\right). \end{array}$$

We summarize the above results as the following theorem. We can use the formula (11) to carry out the gradient descent method for ‖*M*^*T*^*M* − *I*‖_*F*_^2^.

**Theorem 1 .**

Assume *M* ∈ *ℝ*^*n*×*n*^ is the doubly block banded Toeplitz matrix corresponding to the one-channel convolution kernel *K* ∈ *ℝ*^*k*×*k*^. If Ω is the set of all indexes (*i*, *j*) such that *m*_*ij*_=*K*_*p*,*q*_, we have

(11) $$\begin{array}{c} \frac{1}{2}\frac{\partial {\left\|M^{T}M-I\right\|}_{F}^{2}}{\partial K_{p,q}}=\sum_{\left(i,j\right)\in \Omega }\left(\sum_{t=1,\ldots ,n}{\left(M^{T}M-I\right)}_{j,t}m_{\text{it}}+\sum_{s=1,\ldots ,n}{\left(M^{T}M-I\right)}_{s,j}m_{\text{is}}\right). \end{array}$$

Theorem 1 provides new insight into how to regularize a convolutional kernel *K* such that singular values of the corresponding transformation matrix are in a bounded interval. We can use the formula (11) to carry out the gradient-type methods. In the future, we can construct other penalty functions to let the transformation matrix corresponding to a convolutional kernel have some prescribed property and calculate the gradient of the penalty function with respect to the kernel as we have done in this paper.

## 3. The Penalty Function and the Gradient for Multichannel Convolution

In this section, we consider the case of multichannel convolution. First, we show the transformation matrix corresponding to multichannel convolution. At each convolutional layer, we have a convolution kernel *K* ∈ *ℝ*^*k*×*k*×*g*×*h*^, and the input *X* ∈ *ℝ*^*N*×*N*×*g*^; element *X*_*i*,*j*,*d*_ is the value of the input unit within the channel *d* at row *i* and column *j*. Each entry of the output *Y* ∈ *ℝ*^*N*×*N*×*h*^ is produced by

(12) $$\begin{array}{c} Y_{r,s,c}={\left(K*X\right)}_{r,s,c}=\sum_{d\in \left\{1,\ldots ,g\right\}}\sum_{p\in \left\{1,\ldots ,k\right\}}\sum_{q\in \left\{1,\ldots ,k\right\}}X_{r-m+p,s-m+q,d}K_{p,q,d,c}, \end{array}$$

where *X*_*i*,*j*,*d*_=0 if *i* ≤ 0 or *i* > *N*, or *j* ≤ 0 or *j* > *N*. By inspection, *vec*(*Y*)=*Mvec*(*X*), where *M* is as follows:

(13) $$\begin{array}{c} M=\left(\begin{array}{cccc} M_{\left(1\right)\left(1\right)} & M_{\left(1\right)\left(2\right)} & \cdots & M_{\left(1\right)\left(g\right)}\\ M_{\left(2\right)\left(1\right)} & M_{\left(2\right)\left(2\right)} & \cdots & M_{\left(2\right)\left(g\right)}\\ \vdots & \vdots & \cdots & \vdots \\ M_{\left(h\right)\left(1\right)} & M_{\left(h\right)\left(1\right)} & \cdots & M_{\left(h\right)\left(g\right)} \end{array}\right), \end{array}$$

and each *B*_(*c*)(*d*)_ ∈ *𝒯*, i.e., *B*_(*c*)(*d*)_ is a *N*^2^ × *N*^2^ doubly block banded Toeplitz matrix corresponding to the portion *K*_:,:,*d*,*c*_ of *K* that concerns the effect of the *d*-th input channel on the *c*-th output channel.

Similar to the proof in Section 2, we have the following theorem.

**Theorem 2 .**

Assume *M* is the structured matrix corresponding to the multichannel convolution kernel *K* ∈ *ℝ*^*k*×*k*×*g*×*h*^ as defined in (13). Given (*p*, *q*, *z*, *y*), if Ω_*p*,*q*,*z*,*y*_ is the set of all indexes (*i*, *j*) such that *m*_*ij*_=*k*_*p*,*q*,*z*,*y*_, we have

(14) $$\begin{array}{c} \frac{\partial {\left\|M^{T}M-I\right\|}_{F}^{2}}{\partial K_{p,q,z,y}}=2\sum_{\left(i,j\right)\in \Omega_{p,q,z,y}}\left(\sum_{t=1,\ldots ,g*N^{2}}{\left(M^{T}M-I\right)}_{j,t}m_{\text{it}}+\sum_{s=1,\ldots ,g*N^{2}}{\left(M^{T}M-I\right)}_{s,j}m_{\text{is}}\right). \end{array}$$

Then the gradient descent algorithm for the penalty function ‖*M*^*T*^*M* − *I*‖_*F*_^2^ can be devised, where the number of channels may be more than one. We present the detailed gradient descent algorithm for the penalty function ‖*M*^*T*^*M* − *I*‖_*F*_^2^ as follows:

## 4. Numerical Experiments

The numerical tests were performed on a laptop (3.0 GHz and 16G Memory) with MATLAB R2016b.

We use *M* to denote the transformation matrix corresponding to the convolutional kernel. The largest singular value and smallest singular value of *M* (denoted as “*σ*_max_(*M*) and *σ*_min_(*M*)), the iteration steps (denoted as “iter”) are demonstrated to show the effectiveness of our method. The efficiency is related to the step size *λ*. According to our experience, the norm of the matrix reshaped from the gradient tensor *G* ∈ *ℝ*^*k*×*k*×*g*×*h*^ in Algorithm 1 decreases as the number of iteration steps becomes larger. Therefore, we can let the step size *λ* be a small number at first and gradually increase *λ*. In our numerical experiments, for Algorithm 1 we use the following dynamic adjustment of step size:

  if (iter <10)
 
*λ*=1*e* − 5;
  elseif (iter <20)
 
*λ*=1*e* − 4;
  else
 
*λ*=1*e* − 3;
  end

Numerical experiments are implemented on extensive test problems. Our method is effective in letting *σ*_max_(*M*) and *σ*_min_(*M*) be approximate to 1. We present the numerical results for some random generated multichannel convolution kernels.

We start from a random kernel with each entry normally distributed on [0,1], i.e., in MATLAB, *K* is generated by the following command.

rng(1):

(15) $$\begin{array}{c} K=\text{randn}\left(k,k,g,h\right). \end{array}$$

**Figure 1:**
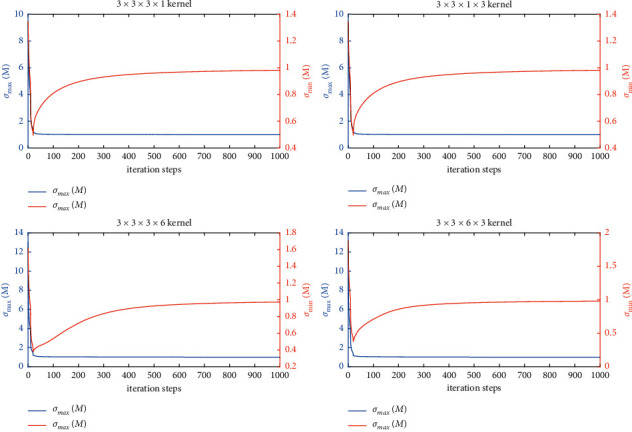
Convergence of *σ*_max_(*M*) and *σ*_min_(*M*) for different kernel sizes.

We consider kernels of different sizes with 3 × 3 filters, namely *K* ∈ *ℝ*^3×3×*g*×*h*^ for various values of *g*, *h*. For each kernel, we use the input data matrix of size 20 × 20 × *g*. We then minimize ℛ_1_(*K*)=‖*M*^*T*^*M* − *I*‖_*F*_^2^ using Algorithm 1 and we demonstrate the beneficial effect of decreasing *σ*_max_(*M*) while increasing *σ*_min_(*M*). We present in Figure 1 the results of 3 × 3 × 3 × 1, 3 × 3 × 1 × 3, 3 × 3 × 3 × 6, and 3 × 3 × 6 × 3 kernels. In the figures, we have shown the convergence of *σ*_max_(*M*) (blue line) on the left axis scale and *σ*_min_(*M*) (red line) on the right axis scale. From Figure 1, we see that at the first about 20 steps, *σ*_max_(*M*) and *σ*_min_(*M*) all decrease and then *σ*_max_(*M*) becomes very close to 1. Then in the following steps *σ*_max_(*M*) become almost unchanged while *σ*_min_(*M*) increase from smaller than 1 to be very close to 1.

We would like to point out, we have used ℛ_1_(*K*)=‖*M*^*T*^*M* − *I*‖_*F*_^2^ to do numerical experiments on other random generated examples, including random kernels with each entry uniformly distributed on [0,1]. The convergence figures of *σ*_max_(*M*) and *σ*_min_(*M*) are similar to the subfigures in Figure 1.

About the running time of Algorithm 1, we have the following remarks. Given a *k* × *k* × *g* × *h* kernel and the input matrix size *N* × *N* × *g*, the gradient tensor *G* ∈ *ℝ*^*k*×*k*×*g*×*h*^ is computed by (14). From the extensive numerical experiments, it is observed that, for a given kernel, when the value of *N* changes, the values of the gradient tensor *G* computed by (14) remain almost unchanged. For example, for the kernel generated by the command “*rng*(1); *K*=randn(3,3,6,3)” “; let *G*^(1)^ denote the gradient tensor computed with *N*=6, and let *G*^(2)^ denote the gradient tensor computed with *N*=64, then each entry *G*_*p*,*q*,*z*,*y*_^(1)^ has at least three significant digits of the corresponding entry *G*_*p*,*q*,*z*,*y*_^(2)^. So in the practical training of neural networks, where *N* could be *N*=64 or even larger for a convolutional layer, we can always compute the gradient tensor *G* with a smaller value of *N*, for example, with *N*=6. From the extensive numerical experiments about different kernels, when *N*=64, *N*=128, and *N*=256, using the gradient tensor *G* computed with *N*=6 to carry out Algorithm 1, the convergence figures of *σ*_max_(*M*) and *σ*_min_(*M*) are similar to the subfigures in Figure 1. For a given 3 × 3 × 64 × 64 kernel, the time for computing the gradient tensor *G* once with *N*=6 is 12.10 seconds on our laptop. For a given 3 × 3 × 5 × 1 kernel, the time for computing the gradient tensor *G* once with *N*=6 is 0.02 seconds on our laptop. The time cost of our regularization method is affordable.

## 5. Conclusions

In this paper, from the viewpoint of linear algebra, we provide the Frobenius norm method to let the convolution be norm preserving, i.e., orthogonal. We let the singular values of the structured transformation matrix corresponding to a convolutional kernel tensor be close to 1. We give the penalty function and propose the gradient decent algorithm for the convolutional kernel tensor. Numerical experiments confirm that this method is effective to modify the singular values of the convolution operations. This mathematical method may cast new light on the training of very deep convolutional neural networks. However, up to now, we only show the method is mathematically feasible and it may be of potential use in the field of deep learning. In the future, further numerical experiments are needed to show the effectiveness of this method during the training of neural networks.

In the future, we will evaluate this method on state-of-the-art architectures like ResNet and DenseNet [26, 27], considering all aspects like recognition rate and computational efficiency. As we know, training a neural network involves many details. It is not easy to see the performance improved while conducting experiments. This will be left as our future work [28].

## Figures and Tables

**Algorithm 1:**
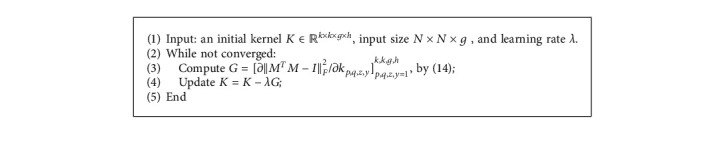
Gradient Descent for ℛ_*α*_(*K*)=‖*M*^*T*^*M* − *I*‖_*F*_^2^.

## Data Availability

Data involved in the paper will be shared upon request. If anybody is interested in the data, please send an email to the corresponding author's email: peichang@cugb.edu.cn. The corresponding author will send the data through email.
